# Complete Genome Sequence of Lopsy, a B1 Cluster Mycobacteriophage

**DOI:** 10.1128/mra.00333-23

**Published:** 2023-06-01

**Authors:** Christopher Ealand, Edith Erika Machowski, Olivia Jacobs, Bavesh Kana

**Affiliations:** a DSI-NRF Centre of Excellence for Biomedical Tuberculosis Research, Faculty of Health Sciences, University of the Witwatersrand, National Health Laboratory Service, Johannesburg, South Africa; DOE Joint Genome Institute

## Abstract

Lopsy is a siphovirus mycobacteriophage that is capable of lytic infection in Mycobacterium smegmatis. It is classified as a subcluster B1 mycobacteriophage and was isolated from soil in Estcourt, South Africa. The 68,542-bp double-stranded DNA genome is circularly permuted, has a GC content of 66.4%, and is predicted to contain 98 genes.

## ANNOUNCEMENT

The emergence of drug-resistant strains of Mycobacterium tuberculosis has highlighted the need for new approaches to tuberculosis treatment. Lytic phage therapies have been applied to a variety of bacterial diseases (1–6); to test this for mycobacterial diseases, identification of lytic phages is important. We isolated Lopsy from moist soil collected between aloes at a petrol station in Estcourt, South Africa (coordinates: −28.924936°, 29.780057°), on 21 July 2022.

**FIG 1:**
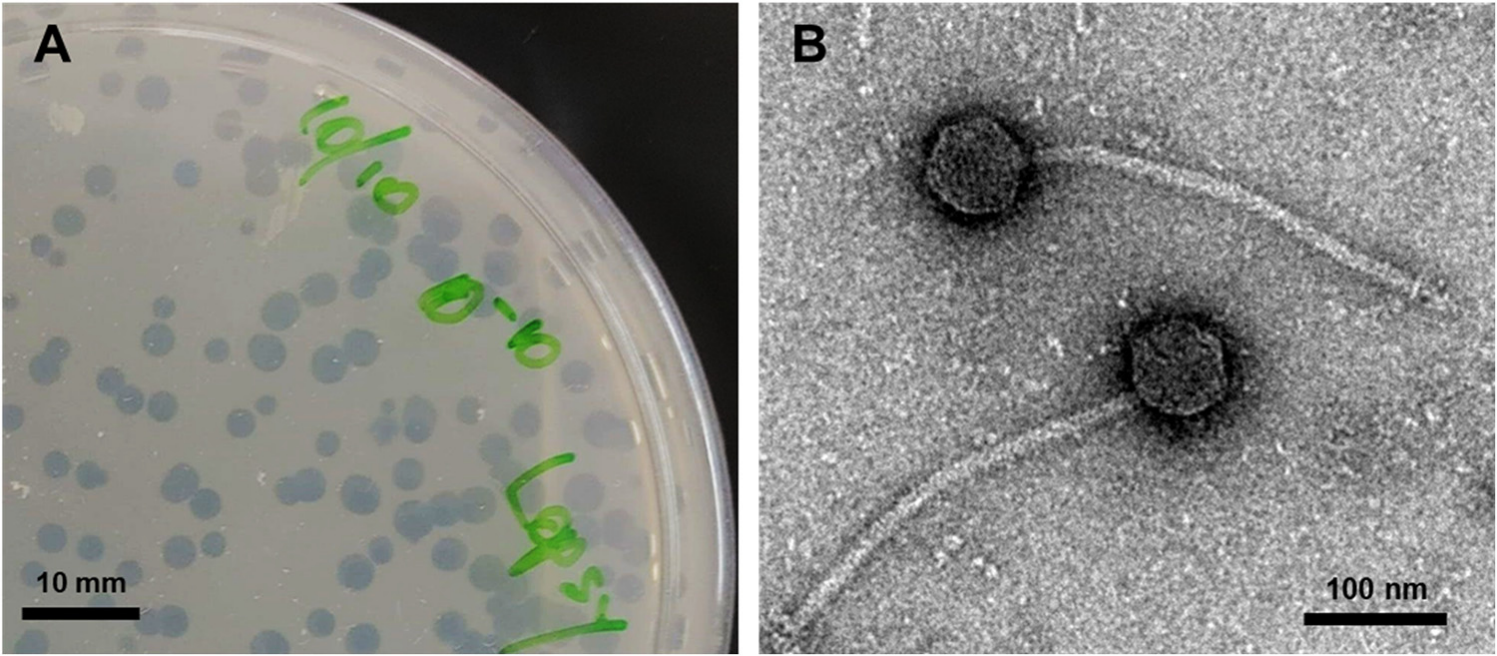
Morphological characterization of mycobacteriophage Lopsy. (A) Petri dish (90 mm) containing solid culture medium (7H10) and bacterial host (Mycobacterium smegmatis mc^2^155) infected with Lopsy. Clear areas show the plaque morphology of Lopsy after incubation for 48 h at 37°C. Plaques appeared to be approximately 2 to 4 mm in diameter and clear, with no visible halo (scale bar = 10 mm). (B) Transmission electron micrograph of virion morphology (stained with 1% uranyl acetate). Lopsy contains an ~65-nm-wide head and a noncontractile tail with a length of ~290 nm (scale bar = 100 nm).

Soil samples were washed with MP buffer (7), and phage particles were purified through a 0.22-μm filter. For infection, 50 μl of filtrate was incubated with stationary-phase Mycobacterium smegmatis mc^2^155 (cultured in 7H9 medium) for 48 h at 37°C (7, 8). Emergent plaques (Fig. 1A) were picked for mycobacteriophage purification as described previously (9). Negative staining transmission electron microscopy showed that Lopsy has a siphovirus morphology, with an icosahedral head diameter and tail length of ~65 nm and ~290 nm, respectively (Fig. 1B).

Genomic DNA was extracted from lysate using the Wizard genomic DNA purification kit (Promega). Library preparation was performed using the NEBNext Ultra II FS kit (New England Biolabs). Briefly, DNA was enzymatically fragmented, size selected (>200 bp) with AMPure XP beads, end repaired, and then ligated with Illumina-specific adapter sequences. The sample was indexed, followed by a second size selection step before sequencing with the Illumina NextSeq 500 platform (300-cycle NextSeq kit). A total of 146,452 reads (2 × 150-bp paired-end reads) were generated and trimmed (Illumina Experiment Manager v1.9 with default settings) before genome assembly using a SPAdes v3.15.4 workflow (10) (https://cab.spbu.ru/software/spades) with the parameter –k (k-mer size) set to 91 and default settings for memory required (–m) and number of threads (–t). A single contig was assembled and checked using Consed v29.0 (11). Lopsy contains a circularly permuted genome of 68,542 bp, with a GC content of 66.4%. The approximate coverage level was 637-fold. Whole-genome nucleotide BLASTn alignments (12, 13) (https://blast.ncbi.nlm.nih.gov) with the standard nonredundant database revealed >99% nucleotide similarity with cluster B1 mycobacteriophages, i.e., Robyn (GenBank accession number MK524526.1), Antonia (GenBank accession number MK279910.1), Aelin (GenBank accession number MW534377.1), Schadenfreude (GenBank accession number MH926060.1), and Manad (GenBank accession number NC_024363.1). Genome annotation was performed using DNA Master v5.23.6 (http://phagesdb.org/DNAMaster), GeneMark v2.5p (14), Glimmer v3.07 (15), Phamerator (https://phamerator.org) (16), HHPRED (https://toolkit.tuebingen.mpg.de/tools/hhpred) with the Pfam-A v35 and NCBI Conserved Domains v3.19 structural/domain databases (17), ARAGORN (18), and tRNAscan-SE v2.0 (19, 20). Default parameters were used for all software except tRNAscan-SE, for which the following modifications were used: sequence source, bacterial; search mode, infernal without HMM; extended options, check Disable pseudo gene checking and check Show primary and secondary structure components to scores; genetic code for tRNA isotype prediction, universal; score cutoff, 17. The Lopsy genome is predicted to contain 98 open reading frames (ORFs) and no tRNAs or transfer-messenger RNAs (tmRNAs). Sixty-three ORFs (~64%) encode hypothetical proteins with unknown functions. The first base of the genome was chosen to match previously published B1 mycobacteriophage sequences. Consistent with this, the Lopsy genome encodes putative structural and assembly genes, including a terminase, an RuvC-like resolvase, capsid and tail-related proteins, and a queuine tRNA-ribosyltransferase. DNA-modifying genes include HNH endonucleases, DNA-binding proteins, helicase, and primase.

### Data availability.

The Lopsy genome sequence is available in GenBank under accession number OQ685900. The raw sequence reads are available in the SRA under BioProject accession number PRJNA935655.
